# Supplementary material for: Learning curve and surgical outcome of robotic assisted colorectal surgery with ERAS program
Source: Sci Rep. 2022 Nov 29;12:20566. doi: 10.1038/s41598-022-24665-w (PMC9709162; doi:10.1038/s41598-022-24665-w)
Supplement: Supplementary file 2 — Supplementary Information 2. [file 41598_2022_24665_MOESM2_ESM.zip › RAL Raw data-Ñ[▒K/136/Patho_ñw╝╨░O▒Kñσ.pdf]

臺中榮民總醫院 臺中市臺灣大道四段1650號 病理部活體組織切片 臨床醫學實驗及病理診斷中心 檢驗(查)報告

[redacted]

Pathologic diagnosis:
1. Lymph node, pelvic, left, dissection --- Unremarkable (0/5).
2. Rectum, cut end, distal, resection --- Chronic inflammation.
3. Rectum, robot-assisted low anterior resection --- Status post-neoadjuvant CCRT with no residual invasive carcinoma. Mucosal ulceration is seen. The bilateral cut ends and perirectal lymph nodes (0/12) are free of tumor.
4. IMA root, dissection --- Unremarkable (0/1).

Ancillary study for diagnosis:
IHC stain for CDX2 done on section AB.

Prognostic and predictive factor:
1. Tumor size: No residual tumor.
2. Depth of invasion: No evidence of residual tumor.
3. Histologic grade: Not applicable.
4. Angiolymphatic invasion: Not identified.
5. Perineural invasion: Not identified.
6. Tumor deposits (discontinuous extramural extension): Not identified.
7. Macroscopic tumor perforation: Not identified.
8. Surgical Margins: a) Proximal Margin: Uninvolved by invasive carcinoma. b) Distal Margin: Uninvolved by invasive carcinoma. c) Radial or Mesenteric Margin: Uninvolved by invasive carcinoma.
9. Lymph node status: Uninvolved (0/18).
10. Extranodal involvement: Not applicable.
11. Tumor regression grading S/P CCRT: Present, no viable cancer cells (complete response, score 0).
12. Type of polyp in which invasive carcinoma arose: Not identified.
13. Additional pathologic findings: Hyperplastic polyp.
14. Pathological TNM Stage: ypT0N0(According to the eighth edition, American Joint Committee on Cancer Staging Guidelines for Tumors).
15. TNM descriptors: y (Post-treatment).

Gross description:
The specimen consists of 1) "left pelvic LN", a piece of tan yellow soft tissue, 4.5x3.5x1.8 cm. 2) distal cut end, a piece of tan mucosa-covered soft tissue, 2x1x0.8 cm. 3) a segment of rectum, 13.5 cm in length and 4 cm in proximal cut end circumference, 6.5 cm in distal circumference, and perirectal soft tissue. An ulcerative mucosal lesion, 1.2 x 1.2 cm, is seen, 1.5 cm from the distal cut end. An erythematous flattened area, 4x2 cm, is noted in the rectal mucosa, 3 cm to proximal cut end. Another erythematous flattened area, 1x1 cm, is noted, 4 cm to distal cut end. The remaining rectum is unremarkable grossly. 4) IMA root, a piece of tan yellow soft tissue, 2x1.5x1 cm. Representative part for section: A-B) all of specimen 1 C) all of specimen 2 D) all of specimen 4 E-F) proximal and distal cut ends in specimen 3 G-L) rectal mucosa with erythematous change M) rectum N-X) perirectal soft tissue and lymph nodes Y-AC) ulcerative mucosal lesion all for sections.
#T-59300_2 #M-09413_2 0 1105 00SP00
#T-C4560_2 #M-09183_2 0 1105 000000

已電子簽章

敬告：本單僅供醫師參考使用，不具任何申請效力！！
請病患妥為保管，若不慎遺失醫院無負個人資料外洩責任！
